# Detection of Alzheimer’s Disease Using Hybrid Meta-ROI of MRI Structural Images

**DOI:** 10.3390/diagnostics14192203

**Published:** 2024-10-02

**Authors:** Xiaoming Zheng, on behalf of the Alzheimer’s Disease Neuroimaging Initiative

**Affiliations:** School of Dentistry and Medical Sciences and Rural Health Research Institute, Charles Sturt University, Wagga Wagga, NSW 2678, Australia; xzheng@csu.edu.au; Tel.: +612-6933-2068

**Keywords:** structural MRI images, Alzheimer’s disease, Meta-ROI, signal detection theory, artificial neural network

## Abstract

Background: The averaged cortical thickness of meta-ROI is currently being used for the diagnosis and prognosis of Alzheimer’s disease (AD) using structural MRI brain images. The purpose of this work is to present a hybrid meta-ROI for the detection of AD. Methods: The AD detectability of selected cortical and volumetric regions of the brain was examined using signal detection theory. The top performing cortical and volumetric ROIs were taken as input nodes to the artificial neural network (ANN) for AD classification. Results: An AD diagnostic accuracy of 91.9% was achieved by using a hybrid meta-ROI consisting of thicknesses of entorhinal and middle temporal cortices, and the volumes of the hippocampus and inferior lateral ventricles. Pairing inferior lateral ventricle dilation with hippocampal volume reduction improves AD detectability by 5.1%. Conclusions: Hybrid meta-ROI, including the dilation of inferior lateral ventricles, outperformed both cortical thickness- and volumetric-based meta-ROIs in the detection of Alzheimer’s disease.

## 1. Introduction

Using structural MRI brain images is one of the established imaging techniques for the diagnosis and prognosis of Alzheimer’s disease (AD) along with tau- and amyloid-PET imaging [1]. The imaging biomarkers of AD are generally defined by using a meta-ROI consisting of several regions of interest in the brain [2]. The regions of interest included in these meta-ROIs are in accordance with the spread of amyloid-β deposition, hyperphosphorylated tau, or neural injury in AD brains [3,4,5,6]. Varied cut-off points for these imaging biomarkers are defined for the dichotomous classification of AD from normal aging [2,6,7].

For structural MRI images, an averaged cortical thickness of ten “AD signature” regions was used by Dickerson et al. [8] as the imaging biomarker. The performance of using cortical thickness in AD diagnosis is slightly better than that of using regional volumes, although the difference is relatively small [9]. Meta-analysis using MRI images with different analytic methodologies suggests that an AUC = 0.86 can be achieved by using a meta-ROI of six regions of cortical thickness [9]. Jack Jr et al. [2] used four regions—entorhinal, inferior temporal, middle temporal, and fusiform—suggesting that including more ROIs in the meta-ROI does not necessarily lead to a higher detectability of AD.

One of the limitations of current MRI-based meta-ROI is the use of either a summation of the regional volumes [10] or an average of the cortical thickness [2], but not both. A summation or average on both volumes and cortical thickness is not practical because they are in different dimensional scales. Although the difference in AD detectability between volumetric and cortical thickness-based meta-ROIs is small [9], a combined or hybrid meta-ROI involving both volume and cortical thickness has not been studied. For a hybrid meta-ROI, the weightings of constituting ROIs can be determined by using an artificial neural network (ANN) [11].

Cardiovascular risk is one of the major factors linked to dementia and Alzheimer’s disease [12,13]. The advantage of using structural MRI images for AD detection is its ability to measure morphologic changes, including the dilation of cerebral ventricles, which was found to be an effective imaging biomarker in differentiating AD from normal controls (NC) [13]. However, cardiovascular factors are not a part of current MRI-based meta-ROIs in AD detection. The main difficulty in using whole cerebral ventricle volumes to differentiate AD from NC is the large overlap of ventricle volume distributions between AD and NC [13]. Regional dilations of cerebral ventricles, such as the temporal horn, which is located next to the hippocampus, were found to be more sensitive in predicting AD progression [14] but are not included in current MRI-based meta-ROIs because various other regions of cerebral ventricles were also dilated in the brains of AD patients [13,15].

In an earlier work, a statistical likelihood ratio procedure from signal detection theory was implemented in the detection of Alzheimer’s disease [10]. Using the likelihood ratio in diagnostic decision-making is termed the ideal observer whose decision criterion, or cut-off point for dichotomous classifications, is at the crossover of the probability distributions of signal and background [10,16]. A cut-off point based on the likelihood ratio is considered the best in differentiating AD from NC [17]. In this work, the AD detectability of selected cortical and volumetric brain regions was first examined using signal detection theory [10,16,18]. Top-performing ROIs were then taken as input nodes to an artificial neural network for AD classification. The AD detectability using dilations of inferior lateral ventricles was found to be almost the same as that of using hippocampal volume reduction. A diagnostic accuracy of AUC = 0.919 was achieved by using a hybrid meta-ROI combining the thicknesses of entorhinal and middle temporal cortices, and the volumes of the hippocampus and inferior lateral ventricles.

## 2. Materials and Methods

From signal detection theory and assuming Gaussian distributions, the detectability index of a signal, $d_{A}$, can be expressed as follows [18]:

(1) $$d_{A}={SNR}_{t}=\frac{{\langle t\rangle }_{1}-{\langle t\rangle }_{2}}{\sqrt{\frac{1}{2}}\sigma_{1}^{2}+\frac{1}{2}\sigma_{2}^{2}}=2{erf}^{-1}\left[2\left(AUC\right)-1\right]$$

where

${SNR}_{t}$ is signal to noise (background) ratio and t is the decision variable.
${\langle t\rangle }_{1,2}$ are the mean values of signal and noise (background).
$\sigma_{1,2}^{2}$ are the standard deviations of the signal and noise (background).
erf is the error function.
AUC is the area under the ROC curve.

If the signal and background are both normally distributed, then [18]

(2) $$AUC=\frac{1}{2}+\frac{1}{2}erf\left(\frac{{SNR}_{t}}{2}\right)$$

**Figure 1:**
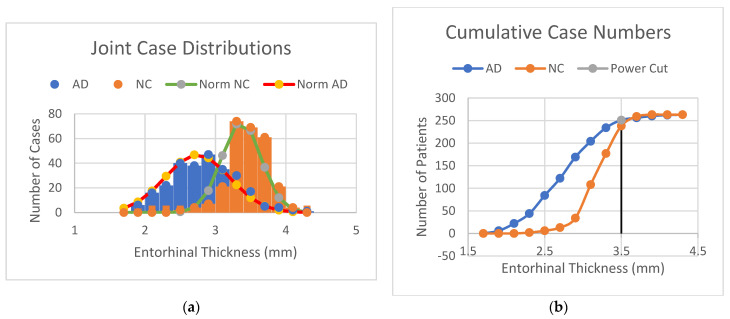
(**a**) Combined case distributions (histograms) of Alzheimer’s disease (AD) and normal controls (NC) on the entorhinal cortical thickness variable. (**b**) Cumulative case numbers of Alzheimer’s disease (AD) and normal controls (NC) on the entorhinal cortical thickness variable.

For the diagnosis of Alzheimer’s disease, the detectability index, $d_{A}$, or area under the ROC curve, AUC, of the individual ROIs can be determined by calculating the mean values and standard deviations of the decision variables such as cortical thickness, regional volumes, or cortical surfaces from AD patients (signal) and CN controls (background). Figure 1a shows the combined probability density distributions of AD and CN, which were constructed using entorhinal cortical thickness as the decision variable, t (imaging biomarker). These probability density functions are derived from the normalized histograms (divided by the total sample number) [10].

An ideal observer makes diagnostic decisions by setting decision criteria or cut-off point at the probability $f\left(AD\right)=f(NC)$, or $y=\ln f\left(AD\right)-\ln f\left(NC\right)=0$ [10,16,18], which is at the intersection of the two probability distributions (Figure 1a). It has the maximum (optimal) diagnostic accuracy, which is the area under the ROC curve, AUC. In Figure 1a, the cut-off point for entorhinal cortical thickness that differentiates AD from NC is at t = 3.04 mm, where the cortical thickness of the entorhinal cortex is used as the decision variable. Human observers, such as radiologists, also use decision criteria in making diagnostic decisions based on the probability density function they established during their specialist training [19]. The decision criteria of human observers can be biased, and their performance is always inferior to that of an ideal observer [20], either by setting the decision threshold higher or lower than the cross-over point of the probability density functions, as shown in Figure 1a.

Figure 1b shows the cumulative case numbers of AD and NC as a function of the entorhinal cortical thickness, or cumulative probability function by normalization. They are sigmoid-shaped functions, and their slopes are inversely proportional to their standard deviations [21]. A higher slope of the cumulative probability function means a smaller standard deviation, which suggests a higher signal detectability [21]. In clinical trials, a sensitivity of 95% with a minimum statistical power of 80% (specificity) is required [22], as shown in Figure 1b. An average diagnostic accuracy of 87.5% or higher is thus expected in clinical practice.

For AD diagnosis, either the thickness of the entorhinal cortex [8] or the volume of the hippocampus [10] may be used as the imaging biomarker or decision variable for AD classifications. A meta-ROI consisting of several regions of interest improves diagnostic accuracy by taking the averaged cortical thickness [2,9] or the total volume of the constituting ROIs [10] as the decision variable. In this work, a hybrid meta-ROI composed of both cortical thickness and regional volumes is used by taking the constituting ROIs as the input nodes to an artificial neural network (multilayer perceptron, SPSS version 27.0 [23]) for AD classification. The ANN uses each of the categories against the rest to determine its diagnostic accuracy, or AUC. Only two categories, or dichotomous classifications, are involved in this work [23].

Seven hundred and eighty-nine T1-weighted MRI images of confirmed definitive AD, MCI, and NC were used in this study. The T1-weighted structural MRI images of AD, MCI, and NC were downloaded from the Alzheimer’s Disease Neuroimaging Initiative (ADNI) [24]. ADNI was launched in 2003 as a public–private partnership, led by Principal Investigator Michael W. Weiner, MD. The primary goal of ADNI has been to test whether serial magnetic resonance imaging (MRI), positron emission tomography (PET), other biological markers, and clinical and neuropsychological assessments can be combined to measure the progression of mild cognitive impairment (MCI) and early Alzheimer’s disease. We used a mixed-gender design without brain size normalization in our study. Using gender as a confounding factor or brain size normalization [2] may or may not improve diagnostic accuracy [10]. We used only one T1-weighted MRI image of each patient for randomness in constructing our probability density functions [10].

**Figure 2:**
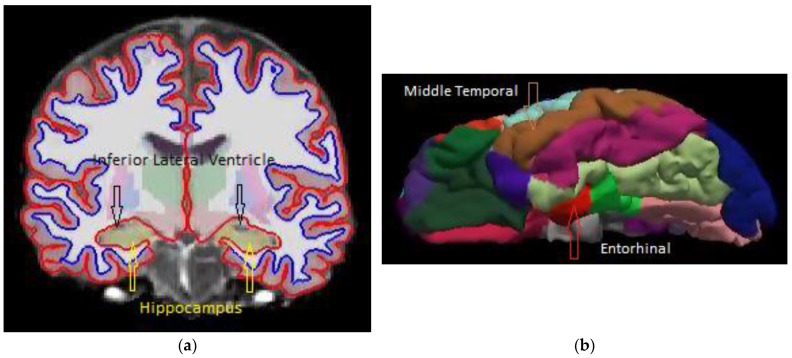
(**a**) A coronal slice of a young healthy subject’s T1-weighted MRI image showing the locations of the hippocampus and inferior lateral ventricles. (**b**) A surface map of the same young healthy subject’s cortical parcellation showing the entorhinal and middle temporal regions.

Regional volume, cortical surface area, and cortical thickness were calculated using FreeSurfer software [25]. FreeSurfer segments brain images into various anatomical regions and cortical areas, as shown in Figure 2. All T1-weighted MRI images in this work were processed using the SPAN computing facility at Charles Sturt University and Australia’s National Computational Infrastructure (NCI) in Canberra. Only selected cortical and volumetric regions from the FreeSurfer outputs are presented in this paper. SPSS statistical package version 27.0 [23] was used to calculate the detectability index or the area under the ROC curve, AUC. The artificial neural network of multilayer perceptron from the SPSS package was used to process the detectability of various combined cortical or volumetric regions.

## 3. Results and Discussion

### 3.1. AD Detectability of Selected Regions of Interest

Table 1 presents the average volume, surface area, and cortical thickness of the selected regions of interest and their standard deviations. The selections of cortical ROIs are based on the meta-ROI from Jack et al. [2], Schwarz et al. [9], and Dickerson et al. [8]. The temporal pole was ranked third in Dickerson’s AD signature regions [8]. The precuneus cortex is a central part of the “default network”, which is believed to be affected in Alzheimer’s disease [26,27,28]. We also include the para-hippocampal cortex because it is a part of hippocampal formation and the medial temporal lobe (MTL) [10]. The volumetric “AD signature” regions include subregions of the MTL, i.e., hippocampus, amygdala, entorhinal cortex, and para-hippocampal cortex [10], as well as the cerebral ventricles [13,14]. Both the whole cerebral ventricles and the inferior lateral ventricle have been reported to be possible biomarkers for AD progression [13,14].

Table 1 shows that most anatomical regions of the brain shrink from NC to AD. The general trend of standard deviations is that there are larger standard deviations (wider spreads of the distributions) of AD than that of NC. Mild cognitive impairment (MCI) appears to be a transitional state from normal controls (NC) to Alzheimer’s disease (AD). The dilation of cerebral ventricle volumes is consistent with the reported values from Nestor et al. [13].

Table 2 shows the percentage changes in volume, surface area, and cortical thickness of the selected regions of interest, along with their detectability index or AUC (the area under the ROC curve). It shows that AD-caused shrinkages vary depending on anatomical regions. The entorhinal cortex shows the largest reduction in both volume (25.2%) and cortical thickness (20.5%) from NC to AD, consistent with the description of “destruction of entorhinal cortex” in AD [5]. However, the AD detectability using entorhinal cortical thickness (AUC = 0.879) is much higher than that using entorhinal volume (AUC = 0.786). On the other hand, the volume reduction of the hippocampus is 19.3%, which is less than that of the entorhinal volumes (25.2%), but its AD detectability of AUC = 0.828 is much higher than that of entorhinal volumes (AUC = 0.786). As shown in Equations (1) and (2), AD detectability not only depends on the difference in mean values of the signal and background but also on their standard deviations. Using the reduction of either regional volume or cortical thickness alone is not a reliable measure for classifying AD from NC.

Cortical surface area is considered a potential biomarker for identifying determinants of cognitive aging differences [29] and for differentiating AD from normal aging [30]. Table 2 shows that the detectability of AD using cortical surface areas is less than that of using either cortical thickness or regional volumes, and it is not sufficient for classifying AD from normal controls [31]. Cortical surface area is correlated with volume to a large extent (R = 0.84) but is independent of cortical thickness (R = −0.17) [29], which is contrary to normal 3D volumetry. These results suggest that cortical thickness and regional volumes can be considered independent variables for AD classification.

**Figure 3:**
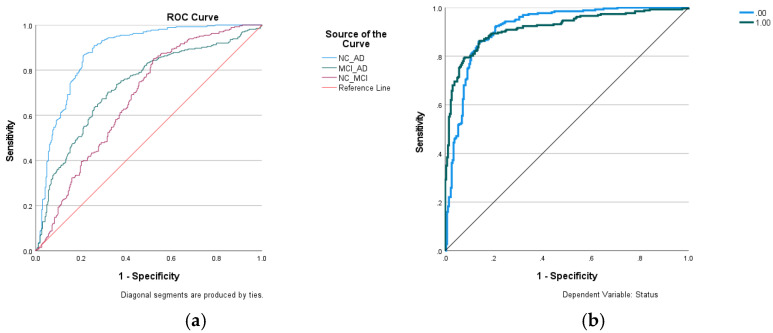
(**a**) ROC curves of AD diagnosis (NC_AD), MCI conversion to AD (MCI_AD), and detection of mild cognitive impairment from normal controls (NC_MCI) using the variable of entorhinal cortical thickness. (**b**) ROC curves of AD (status = 1) diagnosis and NC (status = 0) identification using artificial neural network (ANN), using hybrid input notes of entorhinal and middle temporal cortical thickness, and hippocampal and inferior lateral ventricle volumes (Figure 4a).

Figure 3a shows the detectability (areas under the ROC curves) for AD diagnosis from NC (NC_AD), MCI conversion to AD (MCI_AD), and the early detection of MCI from NC (NC_MCI) using the decision variable of entorhinal cortical thickness. The highest predictability of MCI conversion to AD is 67.6%, and early detection of MCI from NC is 73.0% (Table 2). These figures are consistent with the literature indicating that the detectability is not high enough to predict conversion from MCI to AD or early detection from NC to MCI using MRI structural images [31]. A different strategy for the early detection of AD, based on the dynamic preservation of entorhinal volume, is currently under study [32].

Table 2 shows that there is a 76.2% dilation of the inferior lateral ventricles, which is more than twice that of the 32.5% dilation of the whole cerebral ventricle from CN to AD. The detectability of AD using inferior lateral ventricle volume is AUC = 0.821, which is almost the same as that of using hippocampal volume (AUC = 0.828). In contrast, the AD detectability of using the total volume of cerebral ventricles is only AUC = 0.693 (Table 2), which is not sufficient for practical usage [31]. The high AD detectability of inferior lateral ventricle is not surprising, as it is adjacent to the hippocampus, as shown in Figure 2a. More importantly, the AD detectability of using both hippocampus (AUC = 0.828) and inferior lateral ventricle (AUC = 0.821) volumes is higher than that of using the thickness of inferior temporal (AUC = 0.798) and fusiform (AUC = 0.789) cortices, which are part of the current meta-ROI being used in AD detection [1,2]. A better detectability of AD would be expected if the temporal and fusiform cortices in cortical thickness-based meta-ROI are replaced by the volumes of the hippocampus and inferior lateral ventricles.

### 3.2. Hybrid Meta-ROI for AD Detection

Table 3 shows the processes of taking high-performing ROIs of Table 2 as input nodes for the artificial neural network (ANN) and their resulting AD detectability index, AUC. The number of inputs is gradually added according to their rankings in their detectability index, AUC (Table 2), and the processes are repeated for cortical thickness and regional volumes separately and combined (hybrid cortical thickness and volumes). For AD diagnosis using cortical thickness only, the highest AUC = 0.907 is achieved by including the entorhinal, middle temporal, inferior temporal, and fusiform cortices, which agrees with the findings of Jack Jr et al. [2]. Adding the middle temporal cortex to the entorhinal cortex improved the diagnostic accuracy by 2.4%, and further inclusions of inferior temporal and fusiform cortices improved very little. For AD diagnosis using only volumetric measures, the highest AUC = 0.906 was also achieved by including the hippocampus, inferior lateral ventricles, amygdala, and the entorhinal and middle temporal cortices. The addition of inferior lateral ventricle dilation to hippocampal volume reduction improved the diagnostic accuracy by 5.1%. The overall maximum achievable diagnostic accuracy does not differ when using meta-ROIs of either cortical thickness or volumetric measures, consistent with the conclusion of Schwarz et al. [9].

**Figure 4:**
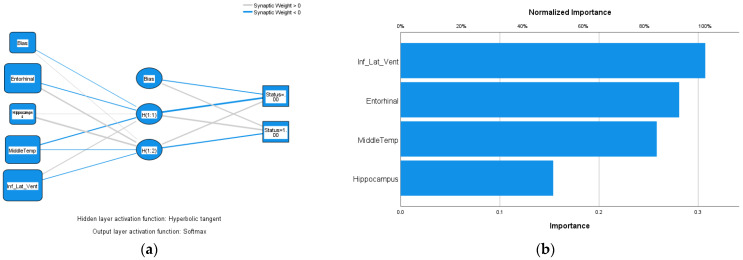
(**a**) The artificial neural network, using input notes of entorhinal and middle temporal cortical thickness and hippocampal and inferior lateral ventricle volumes, achieved the highest AUC = 0.919. (**b**) Normalized importance (weighting) of the four input notes of (**a**).

Similarly, for hybrid inputs into the ANN, both high-performing cortical and volumetric regions were added gradually according to their rankings in AD detectability, as shown in Table 3. The highest AUC = 0.919 was achieved by including entorhinal cortical thickness, hippocampus volume, middle temporal cortical thickness, and inferior lateral ventricle volume. Further inclusion of the amygdala did not improve AD diagnostic accuracy. The ROC curve of the highest AUC is shown in Figure 3b. Figure 4a shows the neural network of the hybrid inputs of the four ROIs that achieved the highest AUC = 0.919. The anatomical regions of these four ROIs are highlighted in Figure 2a,b and Figure 4b, which show the normalized importance (weightings) of the four hybrid input notes. The inferior lateral ventricle carries the highest weighting in the network, as shown in Figure 4b. It states that the dilation of the inferior lateral ventricles is one of the important factors in AD detection and should be a part of the meta-ROI for the diagnosis and prognosis of Alzheimer’s disease. The inclusion of the inferior lateral ventricles in the meta-ROI is consistent with the consensus that cardiovascular risk is one of the major factors of Alzheimer’s disease [12].

## 4. Conclusions

A hybrid meta-ROI combining ROIs of both cortical thicknesses and regional volumes including the dilation of the inferior lateral ventricles outperforms either cortical thickness- or volumetric-based meta-ROIs in AD detection. A diagnostic accuracy of AUC = 0.919 has been achieved by using a hybrid meta-ROI consisting of the thickness of entorhinal and middle temporal cortices and the volume of the hippocampus and inferior lateral ventricles. The dilation of the inferior lateral ventricle is more than twice that of the entire cerebral ventricles in AD. It is one of the important factors affecting Alzheimer’s disease and should be a part of the meta-ROI for the diagnosis and prognosis of AD using structural MRI images. The cut-off point established by an ideal observer of signal detection theory is optimal in dichotomous classifications of Alzheimer’s disease.

## 5. Patents

The author is considering a patent application at present.

## Figures and Tables

**Table 1:** Total volumes (mm^3^) ± standard deviation, surface areas (mm^2^) ± standard deviation, and average cortical thickness (mm) ± standard deviation of the selected regions of interest.

	AD	MCI	NC
Entorhinal			
Volume	3036.7 ± 813.7	3617.2 ± 868.3	3912.4 ± 761.2
Surface	788.5 ± 160.1	820.4 ± 159.8	827.7 ± 157.8
Thickness	2.743 ± 0.458	3.110 ± 0.463	3.370 ± 0.278
Fusiform			
Volume	15,480.3 ± 2609.8	16,774.4 ± 2534.8	17,653.6 ± 2245.0
Surface	5443.9 ± 795.5	5690.3 ± 691.9	5759.1 ± 677.4
Thickness	2.462 ± 0.205	2.563 ± 0.218	2.658 ± 0.134
Inferior temporal			
Volume	16,736.9 ± 3092.3	18,950.8 ± 3277.3	19,502.5 ± 2855.8
Surface	5609.5 ± 940.0	6025.3 ± 915.6	6115.3 ± 858.4
Thickness	2.496 ± 0.188	2.623 ± 0.193	2.685 ± 0.123
Middle temporal			
Volume	17,415.8 ± 3065.2	19,058.1 ± 3087.8	20,264.1 ± 2883.2
Surface	5744.3 ± 884.4	6053.4 ± 791.3	6143.3 ± 820.2
Thickness	2.498 ± 0.200	2.613 ± 0.201	2.717 ± 0.123
Temporal pole			
Volume	4305.1 ± 856.1	4518.3 ± 730.5	4787.0 ± 650.7
Surface	880.5 ± 135.0	902.1 ± 113.3	908.7 ± 108.0
Thickness	3.211 ± 0.385	3.359 ± 0.360	3.552 ± 0.258
Precuneus			
Volume	15,656.2 ± 2500.8	16,362.9 ± 2493.1	17,326.8 ± 2404.8
Surface	7025.9 ± 874.2	7168.3 ± 827.4	7181.2 ± 954.4
Thickness	2.105 ± 0.174	2.163 ± 0.185	2.265 ± 0.150
Para-hippocampal			
Volume	3350.8 ± 625.6	3555.4 ± 596.3	3834.9 ± 518.2
Surface	1199.7 ± 148.4	1218.4 ± 220.3	1229.9 ± 132.0
Thickness	2.409 ± 0.304	2.537 ± 0.323	2.690 ± 0.237
Hippocampus			
Volume	5962.1 ± 1025.3	6559.2 ± 1049.2	7238.4 ± 870.0
Amygdala			
Volume	2340.9 ± 548.1	2594.8 ± 542.8	2957.1 ± 476.6
Inferior Lateral Ventricle			
Volume	3348.5 ± 2440.2	2279.1 ± 1651.6	1500.5 ± 1066.1
Total Ventricle			
Volume	57,312.7 ± 27,368.6	49,605.5 ± 24,277.5	41,296.2 ± 20,535.1

**Table 2:** Detectability index, AUC (area under the ROC curve), and percentage change of the selected regions of interest.

	NC → AD		MCI → AD		NC → MCI	
	AUC	%	AUC	%	AUC	%
Cortical Thickness						
Entorhinal	0.879	−20.5	0.676	−12.5	0.730	−8.02
Middle Temp	0.832	−8.40	0.675	−4.50	0.653	−3.90
Inferior Temp	0.798	−7.30	0.695	−4.96	0.593	−2.34
Fusiform	0.789	−7.66	0.652	−4.02	0.628	−3.64
Temporal Pole	0.773	−10.1	0.618	−4.51	0.668	−5.59
Para-hippocampal	0.764	−11.0	0.623	−5.18	0.638	−5.85
Precuneus	0.754	−7.32	0.593	−2.72	0.656	−4.61
Cortical Surface						
Entorhinal	0.582	−4.85	0.566	−3.97	0.515	−0.89
Middle Temp	0.593	−6.71	0.614	−5.24	0.523	−1.47
Inferior Temp	0.665	−8.63	0.632	−7.15	0.531	−1.48
Fusiform	0.624	−5.63	0.603	−4.43	0.516	−1.20
Temporal Pole	0.569	−3.15	0.561	−2.42	0.502	−0.73
Para-hippocampal	0.571	−2.49	0.526	−1.55	0.547	−0.94
Precuneus	0.543	−2.19	0.548	−2.01	0.496	−0.18
Regional Volume						
Hippocampus	0.828	−19.3	0.661	−9.54	0.790	−9.85
Inf-Lat Ventricle	0.821	76.2	0.669	38.0	0.676	41.2
Amygdala	0.805	−23.3	0.632	−10.3	0.691	−13.1
Entorhinal	0.786	−25.2	0.693	−17.5	0.590	−7.84
Middle Temp	0.752	−15.1	0.652	−9.00	0.604	−6.13
Para-hippocampal	0.736	−13.5	0.600	−5.93	0.641	−7.56
Total ventricle	0.693	32.5	0.584	14.4	0.616	18.3
Precuneus	0.680	−10.1	0.572	−4.41	0.609	−5.72

**Table 3:** ANN input nodes and AD detectability index, AUC (area under the ROC curve).

Cortical Thickness	Input Nodes	AUC	Volume	Input Nodes	AUC
Entorhinal (E), Middle Temporal (M), Inferior Temporal (I), Fusiform (F), Temporal Pole, (T), Para-hippocampal (PH), Precuneus (P).	E	0.879	Hippocampus (H), Inf-Lat Ventricle (IV), Amygdala (A), Entorhinal (E), Middle Temporal (M), Para-hippocampal (PH), Precuneus (P).	H	0.828
	E + M	0.903		H + IV	0.879
	E + M + I	0.901		H + IV + A	0.879
	E + M + I + F	0.907		H + IV + A + E	0.897
	E + M + I + F + T	0.906		H + IV + A + E + M	0.906
	E + M + I + F + T + PH	0.904		H + IV + A + E + M + PH	0.902
	E + M + I + F + T + PH + P	0.906		H + IV + A + E + M + PH + P	0.895
Hybrid					
Cortical Thickness (Thk) and Volume (Vol)			Input Nodes		AUC
Entorhinal (E-Thk), Hippocampus (H-Vol), Middle Temporal (M-Thk), Inf-Lat Ventricle (IV-Vol), Amygdala (A-Vol).			(E-Thk) + (H-Vol)		0.895
			(E-Thk) + (H-Vol) + (M-Thk)		0.911
			(E-Thk) + (H-Vol) + (M-Thk) + (IV-Vol)		0.919
			(E-Thk) + (H-Vol) + (IV-Vol) + (M-Thk) + (A-Vol)		0.914

## Data Availability

The original image data were downloaded from the Alzheimer’s Disease Neuroimaging Initiative (https://adni.loni.usc.edu/ (accessed on 15 January 2020)). The processed data is available upon request from the author.
